# MicroRNA-194 Modulates Glucose Metabolism and Its Skeletal Muscle Expression Is Reduced in Diabetes

**DOI:** 10.1371/journal.pone.0155108

**Published:** 2016-05-10

**Authors:** Celine Latouche, Alaina Natoli, Medini Reddy-Luthmoodoo, Sarah E. Heywood, James A. Armitage, Bronwyn A. Kingwell

**Affiliations:** 1 Baker IDI Heart and Diabetes Institute, Melbourne, Victoria, Australia; 2 School of Medicine (Optometry), Deakin University, Waurn Ponds, Victoria, Australia; University of Birmingham, UNITED KINGDOM

## Abstract

**Background:**

The regulation of microRNAs (miRNAs) at different stages of the progression of type 2 diabetes mellitus (T2DM) and their role in glucose homeostasis was investigated.

**Methods:**

Microarrays were used to assess miRNA expression in skeletal muscle biopsies taken from healthy individuals and patients with pre-diabetes or T2DM, and insulin resistant offspring of rat dams fed a high fat diet during pregnancy.

**Results:**

Twenty-three miRNAs were differentially expressed in patients with T2DM, and 7 in the insulin resistant rat offspring compared to their controls. Among these, only one miRNA was similarly regulated: miR-194 expression was significantly reduced by 25 to 50% in both the rat model and in human with pre-diabetes and established diabetes. Knockdown of miR-194 in L6 skeletal muscle cells induced an increase in basal and insulin-stimulated glucose uptake and glycogen synthesis. This occurred in conjunction with an increased glycolysis, indicated by elevated lactate production. Moreover, oxidative capacity was also increased as we found an enhanced glucose oxidation in presence of the mitochondrial uncoupler FCCP. When miR-194 was down-regulated *in vitro*, western blot analysis showed an increased phosphorylation of AKT and GSK3β in response to insulin, and an increase in expression of proteins controlling mitochondrial oxidative phosphorylation.

**Conclusions:**

Type 2 diabetes mellitus is associated with regulation of several miRNAs in skeletal muscle. Interestingly, miR-194 was a unique miRNA that appeared regulated across different stages of the disease progression, from the early stages of insulin resistance to the development of T2DM. We have shown miR-194 is involved in multiple aspects of skeletal muscle glucose metabolism from uptake, through to glycolysis, glycogenesis and glucose oxidation, potentially via mechanisms involving AKT, GSK3 and oxidative phosphorylation. MiR-194 could be down-regulated in patients with early features of diabetes as an adaptive response to facilitate tissue glucose uptake and metabolism in the face of insulin resistance.

## Introduction

MicroRNAs (miRNAs) are an abundant class of small, non-coding RNAs approximately 22 nucleotides long that have emerged as key regulators of gene expression through translational inhibition and reduction in mRNA stability [1, 2]. They have been implicated in the regulation of numerous biological processes in different tissues. Intensive research in recent years has focused on the identification of miRNAs involved in pathophysiology [3, 4]. A growing number of miRNAs are now implicated in the development of type 2 diabetes mellitus (T2DM) [5] and the regulation of glucose and lipid metabolism [6–8].

Type 2 diabetes constitutes one of the greatest pandemics of our time, with 387 million people currently diagnosed, and 592 million people predicted to be affected by 2035 (IDF Diabetes Atlas 2014). The rapid increase in T2DM over recent decades has been caused by the interaction of genetic susceptibility and environmental factors, such as unhealthy diet and sedentary lifestyle. Indeed, there is now strong evidence that altering the intrauterine environment can result in changes in fetal development, thereby rendering an individual more likely to develop T2DM even when adult risk factors are not present [9, 10]. Progressive impairment of glucose homeostasis is also noted in many models of maternal diet-induced obesity, including in chow fed offspring [11–13].

Skeletal muscle is the major site for postprandial, insulin-stimulated glucose uptake and there is evidence that adult muscle structure and function are affected by an adverse intrauterine environment. Muscle atrophy and intramuscular lipid accumulation and metabolic adaptations normally associated with the onset of insulin resistance are observed in rat offspring exposed to an obesogenic (cafeteria) diet in early life [14]. We previously showed that maternal overnutrition, in the absence of obesity prior to conception, programs insulin resistance in the adult rat offspring fed a normal chow diet from birth. This insulin resistance was associated with alteration in skeletal muscle gene expression related to cytokine activation, inflammation and mitochondrial dysfunction [15]. This occurred prior to any onset of obesity and without consumption of a diet rich in fat or sugars.

The current study aimed to address the hypothesis that key miRNAs in skeletal muscle are regulated early in the development of insulin resistance and involved in glucose homeostasis, affecting long-term health. We thus examined miRNA signatures in skeletal muscle of programmed insulin resistant rats, before the onset of obesity and in the absence of traditional risk factors, and unmedicated individuals who had been newly diagnosed with either pre-diabetes or type 2 diabetes. We identified miR-194 as being commonly down-regulated in humans and rats. Moreover, this miRNA was also decreased in a well established animal model of insulin resistance (high fat diet). The consistency of our findings across models and species at different stages of metabolic disease provided a strong rationale to examine the function of this unique microRNA. We thus investigated the effects of this down-regulation on glucose homeostasis and signaling pathways *in vitro*.

## Materials and Methods

### Human samples

Males with pre-diabetes (PD, n = 5) or type 2 diabetes mellitus (T2DM, n = 6) participated in the study along with age and sex-matched healthy volunteers (H, n = 5). The study was approved by the Alfred Hospital Ethics Committee and performed in accordance with the Declaration of Helsinki. All participants provided written, informed consent. Pre-diabetic and diabetic status were confirmed with the use of standard criteria (PD: fasting plasma glucose between 6.1 and 6.9 mmol/L or a 2-hour blood glucose level between 7.8 and 11 mmol/L, T2DM: fasting plasma glucose >7.1 mmol/L or a 2-hour blood glucose level of >11.1 mmol/L, after a 75-g oral glucose load [oral glucose tolerance test]). All the patients were unmedicated. Diet and physical activity were not controlled nor recorded during the study. However, participants were advised to abstain from alcohol, caffeine and moderate-vigorous exercise for the 24 hours prior to biopsy which was performed after an overnight fast.

Skeletal muscle biopsies were obtained from the *vastus lateralis* of fasted participants using standard aseptic technique and local anesthesia. In brief, a ~7 mm skin incision was made, and the fascia opened. A side cutting muscle biopsy needle was passed through the incision to obtain ~100mg of muscle tissue under suction. All biopsies were cleaned then snap frozen in liquid nitrogen and subsequently stored at -80°C until further analysis.

### Animals

Animals were provided humane care in line with the “Guide for the Care and Use of Laboratory Animals” (NIH publication 86–23, 1985). Animal experiments were approved by the Alfred Medical Research and Education Precinct Animal Ethics Committee and conducted in strict accordance with the National Health and Medical Research Council (NHMRC) of Australia Guidelines for Animal Experimentation.

#### Rat offspring of chow or high fat-fed dams

Animal husbandry and experimental diet details have been previously published [15]. Briefly, female Sprague-Dawley breeder rats were fed either a standard chow diet (C, 7% total fat– 0.5% saturated fat, 10% sucrose wt/wt, 16.1 MJ/Kg; AIN93G, Specialty Feeds, Glen Forest, WA, Australia) or a diet rich in saturated fat (sourced mainly from animal lard) and sucrose (HF, 23.5% total fat– 9.83% saturated fat, 20% sucrose wt/wt, 19.6MJ/Kg; SF08-023, Specialty Feeds) for the 3 weeks prior to mating and throughout pregnancy and lactation. Male offspring of control (n = 6) and HF (n = 5) dams were weaned at postnatal day 21 and maintained on a standard chow diet (*ad libitum*) until they reached 12 months. Blood was collected via cardiac puncture and glucose determined by handheld glucometer (Accu-Chek Roche, Castle Hill, NSW, Australia), plasma assayed for insulin by ELISA (Kit 90010, Crystal Chem, Downers Grove, IL, USA).

#### High fat-fed mice

A high fat diet (HFD) was chosen as a model to induce skeletal muscle insulin resistance, as it has been well-characterized in C57BL/6 mice [16–18]. Male C57BL/6 mice aged 8 weeks were fed either a standard chow diet (8% total fat, 14 MJ/kg; Specialty Feeds) or a high fat diet (HFD, 43% total fat (lard), 19 MJ/kg; SF04-001, Specialty Feeds) for a subsequent 8 weeks (n = 9–10 per group) as this duration of diet robustly induces skeletal muscle insulin resistance [18]

For tissue collection, all animals (i.e. rat offspring of chow or high fat-fed dams, and high fat-fed mice) were anaesthetized using inhaled isofluorane. Skeletal muscle (soleus) was collected from the hind limb and immediately snap frozen in liquid nitrogen and stored at -80˚C for further analysis.

### RNA extraction and miRNA microarray

Total RNA (including miRNAs) was extracted from frozen tissues using the TRIZOL reagent (Invitrogen, Mount Waverley VIC, Australia) following the manufacturer’s recommended protocol. Concentration and purity of the total RNAs were assessed by spectrophotometer (Nanodrop 1000, Nanodrop Technologies, Wilmington, DE, USA) and RNA integrity was verified using an Agilent 2100 bioanalyzer (Agilent Technologies, Mulgrave, VIC, Australia).

MiRNA microarrays were performed at the Ramaciotti Centre for Genomics (University of New South Wales, NSW, Australia) using Agilent arrays and following manufacturer’s instructions. Briefly, Cy3-labeled RNA was generated using the Agilent’s miRNA Complete Labeling and Hyb Kit with a sample input of 100 ng of total RNA. Hybridization of labeled miRNA to either 8x60K Human or 8x15K Rat miRNA microarray slides (based on miRBase release 16.0) was for 20 hours at 55°C on a rotator at 20 rpm. The slides were then washed and scanned with an Agilent microarray scanner. Extracted data were analyzed with GeneSpring GX7.3 expression analysis software (Agilent Technologies). In this study, Human miRNA microarray slides contained 1205 human miRNAs, and rat miRNA microarray slides contained 679 mature rat miRNAs. The data discussed in this publication have been deposited in the National Center for Biotechnology Information (NCBI)'s Gene Expression Omnibus (GEO) and are accessible through GEO Series accession number GSE68226 (http://www.ncbi.nlm.nih.gov/geo/query/acc.cgi?acc=GSE68226). Analysis of the miR microarray data was performed in GeneSpring GX7.3 expression analysis software. Differentially expressed miRs were identified following a one way-ANOVA with Student-Newman-Keuls *post hoc* tests in human samples, and unpaired Student t-tests in rat samples. We then aligned data from both species in order to identify miRNAs that were commonly deregulated.

### Cell culture

L6 rat myoblast cells were maintained in Dulbecco's modified Eagle's medium (DMEM) containing 5mM glucose and supplemented with 10% fetal bovine serum and 1% penicillin/streptomycin. Once cells reached confluence, the medium was replaced with DMEM supplemented with 2% horse serum and 1% penicillin/streptomycin to promote myoblast differentiation into myotubes.

### Transfection of miR-194 inhibitor

The amount of miRNA inhibitor needed to efficiently down regulate a target gene or inhibit miRNA function can vary greatly, depending on the miRNA, the cell line, and the chosen analysis method. To determine the optimal concentration, experiments using varying miR-194 inhibitor concentrations (5–100 nM) were performed. A concentration of 100 nM proved optimal, giving robust and consistent efficacy inducing a 35 fold decrease in miR-194 expression in the cells (qPCR, data not shown).

Fully differentiated L6 cells were transfected with either a miR-194 inhibitor or a negative control (Applied Biosystems, #MH10004 and #4464076) using Lipofectamine RNAiMAX (Invitrogen) following the manufacturers’ recommended protocol. Briefly, L6 cells, seeded on 12-well plates, were incubated with 200μl of opti-MEM supplemented with 2% horse serum, 100 nM of miR inhibitor or control (1μl of a 20 μM stock) and 1% of the transfection agent. After 6 h, the supernatant was replaced with fresh medium and cultured for another 42 h. Cells were then either harvested and subjected to RT-qPCR and Western blot analyses or used for the functional assays described below.

Cell viability and integrity was monitored during the transfection period. Of note, all the functional assays were also performed in untransfected cells and in presence of the transfection agent (i.e. lipofectamine) only. No differences were observed between untransfected cells, lipofectamine only and miR negative control.

### miR quantitative real-time PCR

MiR-194 levels were assessed in human and rodent tissues, and in transfected L6 cells using Taqman microRNA assays (Applied Biosystems, Carlsbad, CA, USA). RNA (2 μg) from the skeletal tissues was reverse transcribed using the specific miR-194 stem-loop RT primers (Applied Biosystems, #000493) and then subjected to quantitative PCR on the Applied Biosystems 7500 Real-Time PCR System. Data were normalized to RNU44 (for human tissues, Applied Biosystems, #001094) or U6 snRNA (for rodent tissues and cells, Applied Biosystems, #001973) and analyzed using the ΔΔCt method [19].

### Basal and insulin-stimulated glucose uptake

Glucose uptake was determined by the 2-deoxy-glucose method. Following overnight serum depletion, cells were pre-treated with or without 100nM insulin for 30 min in DMEM containing 5 mM glucose, before a 20 min-treatment with or without insulin in glucose-free media. Following treatment, glucose uptake was assayed by incubating cells with 2-deoxy-[^3^H]-d-glucose (1 μCi/ml, 26.2 Ci/mmol) for 10 min. Cell-associated radioactivity was determined by lysing the cells with 0.3M NaOH, followed by liquid scintillation counting and protein determined with the Pierce BCA Protein Assay (ThermoScientific, Rockford, IL, USA). Nonspecific uptake was assessed with the use of cytochalasin-B (10 μmol/L), a vesicle transport inhibitor, and was subtracted from total uptake.

### Basal and insulin-stimulated glycogen synthesis

*In vitro* rates of basal and insulin-stimulated glucose utilization via incorporation into glycogen were determined in L6 cells after miR transfection. L6 myotubes were serum deprived overnight in DMEM (5mM glucose, 1% penicillin/streptomycin) and washed twice with warm PBS prior to incubation at 37 °C with or without 100 nm insulin, and 0.8μCi/ml [U^14^C]-d-glucose for 1 hour. The incubation was terminated by three washes with ice-cold PBS prior to lysis in 1M KOH. Cellular glycogen was precipitated from lysates using glycogen (25mg/ml), saturated Na_2_SO_4_, and ice-cold absolute EtOH, and incubated overnight at -80 °C. The following day, samples were homogenized by sonication in ice water and associated radioactivity was determined by liquid scintillation counting. Protein concentration was determined with the Pierce BCA Protein Assay (ThermoScientific).

### Basal and insulin-stimulated lactate production

*In vitro* rates of basal and insulin-stimulated glycolysis were determined in L6 cells after miR transfection via measure of lactate production. L6 myotubes were serum deprived for 4 hours in DMEM (5mM glucose, 1% penicillin/streptomycin) and washed twice with warm PBS prior to a 2 hour-incubation at 37 °C with or without 100 nm insulin [20]. Media was then removed and an acid extraction was carried out. Lactate production was determined enzymatically using L-Lactate (Sigma) to generate standard curve.

### Glucose oxidation

L6 cells were plated in 12-well plates and serum starved overnight prior to 1 hour pre-incubation with 1mM glucose-Krebs buffer. Krebs was removed and L6 were treated with saline or FCCP in Krebs buffer containing 1μCi/mL [^14^C]-glucose for 2 hours. [^14^CO_2_] produced during glucose oxidation was collected using a CO_2_ trapping method. 250μL of buffer was removed and carefully added to a 20mL scintillation vial containing a 1.7mL tube with a 500μL tube inside. 300μL of 1M NaOH was added into the 500μL tube and an equal volume of 1M acetic acid was added to the scintillation vial prior to incubation in a shaking incubator for 2 hours at 37°C. Shaking allows the [^14^CO_2_] to be released and captured in the NaOH. Following incubation, the NaOH containing 500μL tube was removed and placed into a separate 5mL scintillation vial and associated radioactivity was determined by liquid scintillation counting. Protein concentration was determined with the Pierce BCA Protein Assay (ThermoScientific).

### Western blots

Muscle samples were homogenized in lysing buffer (20mM HEPES, 2M EDTA, 50mM NaF, 5mM Na4P2O7, 1% NP40, 1mM Na3VO4, 1mM DTT, 0.05% SDS) containing a protease inhibitor cocktail (Roche Diagnostics, Meylan, France). The homogenates were centrifuged at 12,000 g for 10 min and the supernatant recovered. Protein content was then determined with the Pierce BCA Protein Assay (ThermoScientific). Proteins were reduced and denatured by boiling lysates in Laemmli buffer at 95°C for 5 min. Lysates were then separated by gradient (4–20%) SDS-polyacrylamide gel electrophoresis (Novex, Carlsbad, CA, USA) and transferred to PVDF membranes (GE Healthcare, Rydalmere, NSW, Australia). Blots were blocked 1 h at room temperature with 5% non-fat dry milk in TBST (20mM Tris-HCl pH 8.0, 135mM NaCl, 0.1% Tween-20). Membranes were then incubated with anti-phosphoAkt(Ser473) (1/2000, #4058, Cell Signaling Technology, Danvers, MA, USA), anti-total Akt (1/2000, #9272, Cell Signaling Technology), anti-phosphoGSK3α/β(Ser21/9) (1/1000, #9331, Cell Signaling Technology, the lower band representing phosphoGSK3β was quantified), and anti-total GSK3β (1/2000, #9315, Cell Signaling Technology)antibodies overnight. After several washes with TBST, membranes were exposed to anti-rabbit horseradish peroxidase-conjugated secondary antibody for 1 hr. Antibody binding was then detected using the ECL Plus reagents (GE Healthcare) and signals were quantified using the Molecular Imager ChemiDoc and Quantity One 1-D analysis software (Bio-Rad Laboratories, Hercules, CA, USA). Beta-actin (#4967, Cell Signaling Technology) was used as an internal protein loading control for the AKT and GSK3β blots. The same membranes were used to detect both the phosphorylated and the total forms of each protein of interest (i.e. AKT and GSK3β) and β-actin. The ratio between the phosphorylated and the total forms of both AKT and GSK3β was then calculated.

The Abcam anti-oxidative phosphorylation complexes (OXPHOS) kit was used to determine the relative levels of the 5 OXPHOS complexes (1/250, #ab110413, Abcam, Waterloo, NSW, Australia). This kit contained a cocktail of 5 antibodies, one each against complex subunit. The samples were heated at 50°C for 5 min then resolved using SDS-PAGE. Membranes were exposed to anti-mouse horseradish peroxidase-conjugated secondary antibody, and signals revealed with different durations of exposure. Due to denaturation conditions that differed from prior analyses, α-tubulin (#2144, Cell Signaling Technology) was measured on different membranes.

### Sample size and statistical analysis

Statistical analyses were performed with SigmaStat software (Systat Software Inc., San Jose, CA, USA) and SPSS software (IBM, Armonk, NY, USA).

Although the number of patients and animals was small, a 25% or greater change in gene expression at p<0.05 was identified with power between 87% (human) and 99% (mice).

For qPCR and western blots, normally distributed data were compared by unpaired *t-*tests or one way-ANOVA with Student-Newman-Keuls *post-hoc* tests as appropriate. Non-normally distributed data were compared by Mann-Whitney rank sum tests or Kruskal-Wallis 1-way ANOVA on ranks with Student-Newman-Keuls *post-hoc* tests as appropriate. Relationships between miR expression assessed by qRT-PCR and the insulin resistance index (HOMA-IR) were assessed using the Pearson or the Spearman correlation coefficient as appropriate. Functional data (glucose uptake, glycogen synthesis, glucose oxidation and lactate production) were analyzed by 2-way ANOVA with Student-Newman-Keuls *post-hoc* tests or Kruskal-Wallis 1-way ANOVA on ranks with Student-Newman-Keuls *post-hoc* tests as appropriate. Results are expressed as mean±SEM unless otherwise indicated. Cell culture data represent a minimum of 4 individual experiments, with 4 technical replicates. A *P* value of <0.05 was considered significant.

## Results

### Phenotypic characteristics (humans and rats)

In this study, we used skeletal muscle biopsies taken from unmedicated, newly diagnosed patients with pre-diabetes or type 2 diabetes and healthy volunteers, and soleus muscle from adult rat offspring from dams fed a high fat diet or chow diet during pregnancy and lactation.

Patients with pre-diabetes and type 2 diabetes were obese, hyperglycemic and/or glucose intolerant and hyperinsulinemic compared to age-matched healthy participants (Table 1).

**Table 1:** Phenotypic characteristics.

**HUMAN**	**Healthy**	**Pre-diabetes**	**Type 2 diabetes**
Age (yrs)	48±3	52±2	53±2
BMI (kg/m^2^)	25±1	34±1*	33±2*
Fasting glucose (mM)	5.2±0.2	6.1±0.2	11.2±1.6*
OGTT 2hrs (mM)	4.9±0.5	8.2±0.4	20.4±1.4*
Fasting insulin (mU/L)	4.0±0.5	11.2±2.0**	12.8±1.8**
HOMA-IR	1.0±0.1	3.4±0.7*	6.2±1.3**
**RAT**	**Control**	**High fat**	***p-value***
Body weight (g)	773±38	782±69	0.91
Glucose (mM)	6.35±0.38	7.35±1.24	0.38
Insulin (mU/L)	40.0±2.5	56.2±6.1	**0.02**
HOMA-IR	11.4±0.9	18.9±4.68	0.09

Data were obtained from healthy volunteers (n = 5), or patients with pre-diabetes (n = 5) or type 2 diabetes mellitus (n = 6) and from rat offspring of control (n = 4) or high fat (n = 6) dams at 1 year of age. Values are expressed as mean ± SEM. *P*-values were determined using 1-way ANOVA followed by Student-Newman-Keuls *post-hoc* tests for human data (*p<0.05 and **p<0.01 vs healthy), or Student’s t-test or Mann-Whitney U test as appropriate for rat data. BMI: body mass index, OGTT: oral glucose tolerance test.

Adult rat offspring from dams fed a HF diet were not overweight though they presented with higher adiposity, and were hyperinsulinemic (Table 1). More detailed characteristics on these programmed insulin resistant rats are published elsewhere [15].

We utilised the exact experimental approach as reported in the study by Turner et al., (i.e same mice age and background, same diet, same diet duration) which robustly found 8 weeks of HFD induced skeletal muscle insulin resistance [18]. We confirmed the HFD increased body weight gain, fat mass, glucose intolerance and hyperinsulinaemia using body composition analysis and an oral glucose tolerance test at 6 weeks of diet intervention (unpublished data). These data replicate the same effects of the HFD reported by Turner et al., and provide evidence skeletal muscle insulin resistance is likely induced in our cohort of mice.

### Differential expression of microRNAs in insulin resistant skeletal muscle from rodents and humans

To identify microRNAs that are regulated early in insulin resistance and in the progression to T2DM, we performed miRNA microarray analysis on the muscle samples. We identified 13 up-regulated and 10 down-regulated miRs in the skeletal muscle of patients with T2DM, along with 2 up-regulated and 5 down-regulated miRs in the insulin resistant offspring of fat-fed rats compared to their controls (Table 2). Among these, one miR was similarly regulated; indeed, miR-194 expression was significantly reduced in both models.

**Table 2:** Differentially expressed microRNAs identified by microarray.

**Differentially expressed microRNAs in human skeletal muscle**			
**Systematic name**	**FC PD vs H**	**FC T2DM vs H**	**p-value**
**Upregulated microRNAs **			
hsa-miR-542-5p	22.64	12.58	0.042
hsa-miR-944	2.19	3.48	0.049
hsa-miR-214	1.85	2.65	0.049
hsa-miR-382	2.88	1.98	0.049
hsa-miR-206	1.25	1.73	0.012
hsa-miR-29b-1	1.31	1.53	0.0001
hsa-miR-489	1.36	1.43	0.004
hsa-miR-3907	1.23	1.31	0.015
hsa-miR-139-5p	1.07	1.28	0.030
hsa-miR-29b	1.05	1.24	0.001
hsa-miR-628-5p	1.19	1.22	0.019
hsa-miR-128	1.35	1.20	0.023
hsa-miR-125b	1.19	1.11	0.009
**Downregulated microRNAs**			
hsa-miR-30d	-1.16	-1.10	0.035
hsa-miR-106b	-1.29	-1.25	0.048
hsa-miR-185	-1.47	-1.41	0.029
hsa-miR-194	-1.60	-1.41	0.018
hsa-miR-4271	-1.20	-1.49	0.042
hsa-miR-3141	-1.45	-1.59	0.006
hsa-miR-4306	-1.59	-1.61	0.037
hsa-miR-363	-1.33	-1.68	0.045
hsa-miR-142-3p	-1.86	-1.86	0.047
hsa-miR-515-5p	-4.45	-8.59	0.003
**Differentially expressed microRNAs in rat skeletal muscle**			
**Systematic name**	**FC HF vs C**		**p-value**
**Upregulated microRNAs **			
rno-miR-582	18.12		0.009
rno-miR-181d	7.70		0.014
**Downregulated microRNAs **			
rno-miR-511	-1.20		0.027
rno-miR-322	-1.22		0.008
rno-miR-425	-1.41		0.044
rno-miR-499	-1.98		0.045
rno-miR-194	-2.88		0.019

Fold-change (FC) in microRNA expression is shown in pre-diabetics (PD) or type 2 diabetics (T2DM) vs healthy participants (H) for human data, and in offspring from high fat-fed dams (HF) vs offspring from chow fed dams (C). *P*-values were determined using 1-way ANOVA followed by Student-Newman-Keuls *post-hoc* tests for human data, or Student’s t-test for rat data.

**Fig 1:**
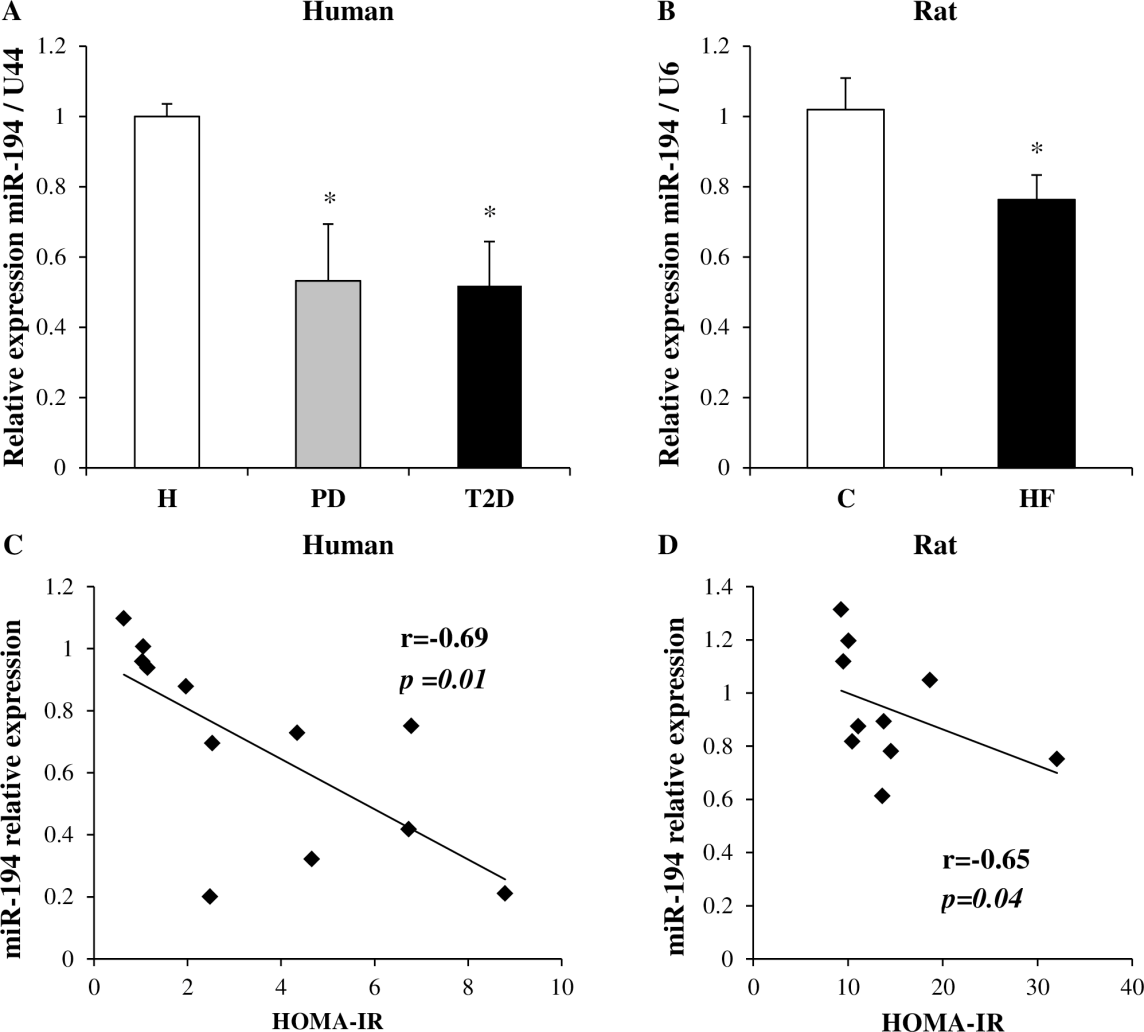
miR-194 expression in the skeletal muscle of human participants and rat offspring and correlations with HOMA-IR. miR-194 expression was validated by qPCR in human (A) and rat samples (B) (n = 4–6 per group). Values are expressed as mean ± SEM. *P*-values were determined using 1-way ANOVA followed by Student-Newman-Keuls *post-hoc* test for human data (*p<0.05 vs healthy), or Student’s t-test for rat data (*p<0.05 vs control). Correlation between miR-194 expression and HOMA-IR in human (C) and rat (D) was assessed using Pearson’s or Spearman’s correlation test as appropriate. R and p-values are indicated on the graphs.

The expression levels of miR-194 were validated by qPCR, demonstrating a 50% reduction in expression in the skeletal muscle of individuals with pre-diabetes and diabetes (Fig 1A) and a 25% decrease in the skeletal muscle of insulin resistant rats (Fig 1B). Moreover, we identified a negative correlation between homeostatic model assessment index of insulin resistance (HOMA-IR, reported in Table 1) and miR-194 expression levels (r = -0.69, p = 0.01 in humans, r = -0.65, p = 0.04 in rats, Fig 1C and 1D), indicating an association of miR-194 with insulin resistance.

**Fig 2:**
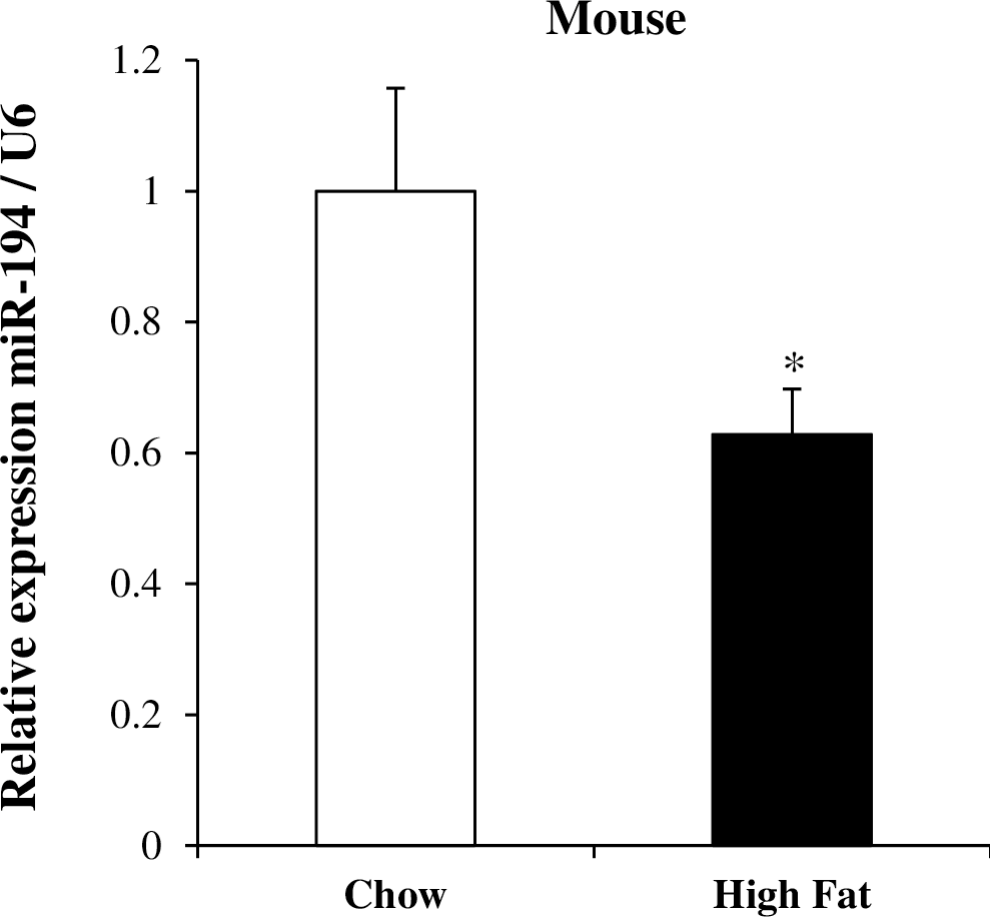
MiR-194 expression in the skeletal muscle of mice fed a high fat diet. MiR-194 expression was measured by qPCR in the skeletal muscle from mice fed a chow or a high fat diet for 8 weeks (n = 9–10 per group). Values are expressed as mean ± SEM. *P*-values were determined using Student’s t-test (*p<0.05 vs chow fed mice).

To further confirm down-regulation of miR-194 in the insulin resistant state, we measured its expression in skeletal muscle from mice fed a HFD for 8 weeks, a well characterized mouse model of insulin resistance [18]. Indeed, we found a 37% decrease in the skeletal muscle of these animals (Fig 2).

### Down-regulation of miR-194 in vitro promotes glucose metabolism

To investigate the functional effect of down-regulation of miR-194 expression in the development of T2DM, we performed *in vitro* functional assays.

**Fig 3:**
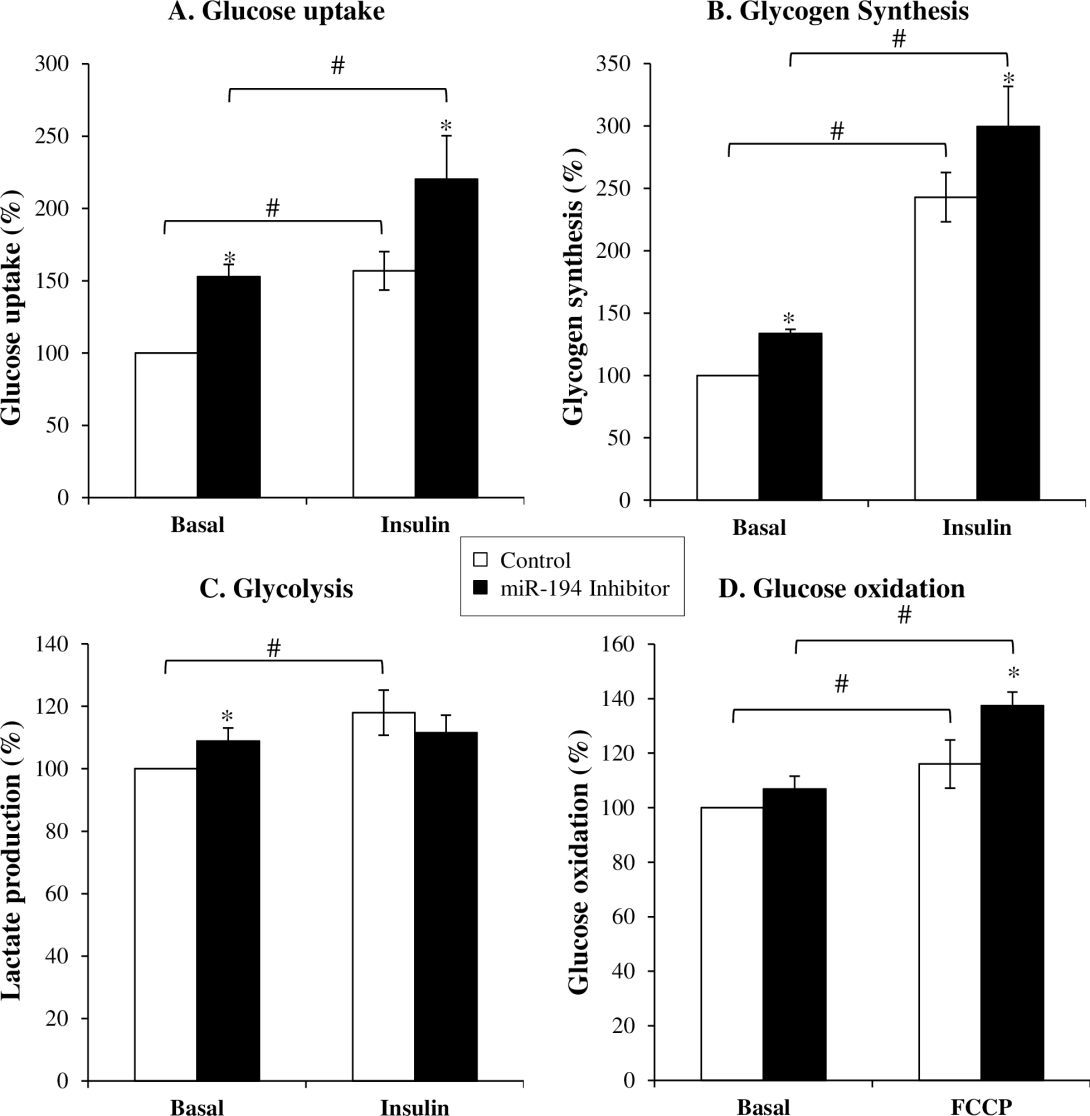
Effect of miR-194 inhibition on glucose homeostasis in L6 cells. Basal or insulin-stimulated glucose uptake (A), glycogen synthesis (B) and lactate production (C) were assayed in L6 cells 48 hours after transfection with a miR-194 inhibitor. Glucose oxidation (D) was also measured in these transfected cells under basal conditions or after treatment with the mitochondrial uncoupler FCCP. Values are expressed as mean ± SEM of 4–6 individual experiments, with 4 technical replicates. *P*-values were determined using 2-way ANOVA (normally distributed data) or Kruskal-Wallis 1-way ANOVA on ranks (non-normally distributed data) followed by Student-Newman-Keuls *post-hoc* tests (* p<0.05 vs. control, # p<0.05 vs. basal).

We firstly assessed basal and insulin-stimulated glucose uptake in the L6 muscle cell line after inhibiting miR-194 expression. Differentiated L6 cells were transfected with either a miR negative control or miR-194 inhibitor. After 48 hours, we measured uptake of the glucose analogue, 2-deoxy-D-glucose. Inhibition of miR-194 induced a 53±8% increase in basal glucose uptake and a 40±20% increase in insulin-stimulated glucose uptake into the muscle cells compared to the transfection control (p<0.05 for both, Fig 3A).

To determine if the differences in glucose uptake contributed to glycogen storage, we then measured glycogen synthesis. Compared to control, miR-194 inhibition increased glycogen synthesis by 34±3% under basal conditions and 23±13% with insulin stimulation (p<0.05 for both, Fig 3B). Additionally we measured lactate production to assess glycolysis. Under basal conditions, we found lactate production was significantly increased following miR-194 inhibition compared to control (9±4%, p<0.05, Fig 3C). When stimulated with insulin, lactate production increased by 18±7% in control cells while there was no further increase in cells transfected with the miR-194 inhibitor (p<0.05, Fig 3C).

To further investigate the fate of this increased glucose uptake in the miR-194 inhibited cells, we measured glucose oxidation. Although there was no effect of miR-194 inhibition on the basal glucose oxidation rate, we found an increased glucose oxidation when the cells were treated with the mitochondrial uncoupler FCCP (Fig 3D).

### Down-regulation of miR-194 in vitro activates the insulin signaling pathway and oxidative phosphorylation

**Fig 4:**
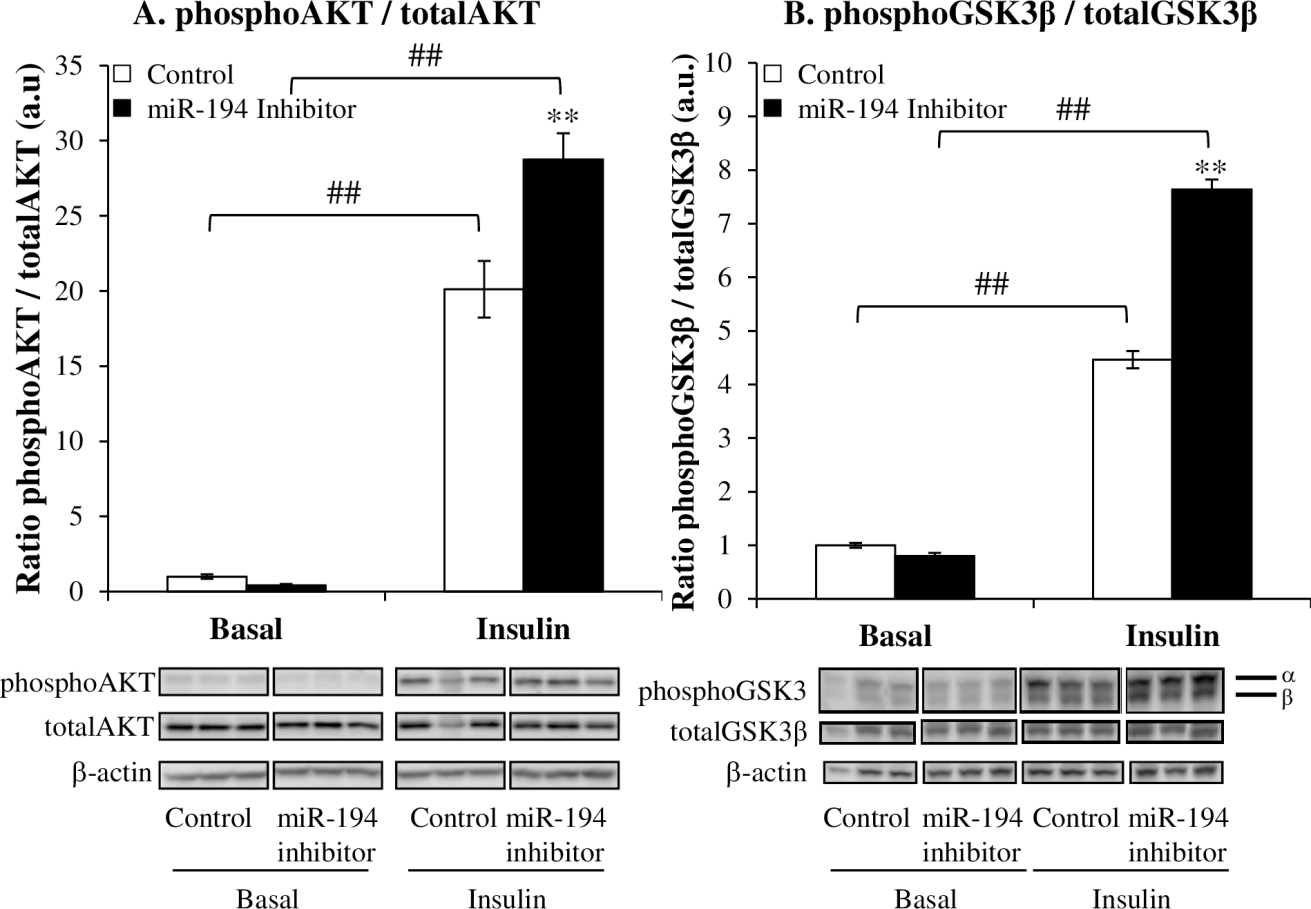
Effect of miR-194 inhibition on AKT and GSK3β expression in L6 cells. Expression of the phosphorylated and total forms of AKT (A) and GSK3β (B) were measured by Western blot in L6 cells 48 hours after transfection with a miR-194 inhibitor. Ratios phosphorylated:total are expressed as mean ± SEM (n = 3 per group). Representative blots are shown below the graphs. *P*-values were determined using 2-way ANOVA (normally distributed data) or Kruskal-Wallis 1-way ANOVA on ranks (non-normally distributed data) followed by Student-Newman-Keuls *post-hoc* tests (* p<0.05 vs. control, # p<0.05 vs. basal).

To address the possible mechanisms by which miR-194 regulates glucose metabolism, we examined components of signaling pathways contributing to glucose metabolism. Phosphorylation of AKT (Ser473) and GSK3β (Ser9), relative to total expression, in response to insulin were increased by 43±9% and 71±4% respectively (p<0.05) in L6 cells transfected with the miR-194 inhibitor (Fig 4). There was no effect on phosphorylation of these proteins by miR-194 inhibition under basal conditions.

**Fig 5:**
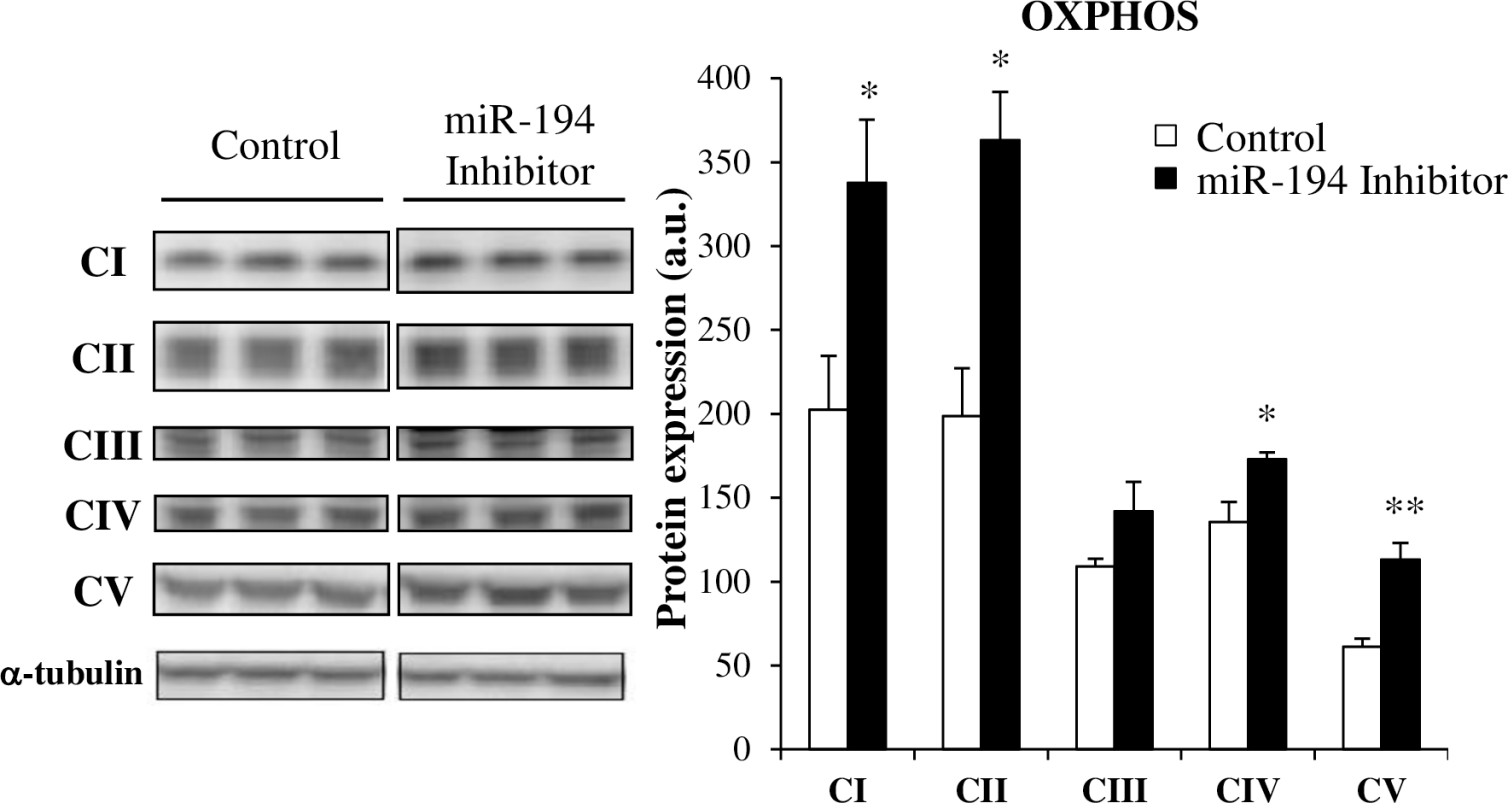
Effect of miR-194 inhibition on protein expression of the oxidative phosphorylation complexes expression in L6 cells. Expression of the oxidative phosphorylation (OXPHOS) complexes I to V of the electron transport chain was measured by western blot in L6 cells 48 hours after transfection with a miR-194 inhibitor. Data are expressed as mean ± SEM (n = 3 per group). Representative blots are shown. *P*-values were determined using t- tests (*p<0.05 and **p<0.01 vs control).

We also examined protein expression of mitochondrial oxidative phosphorylation (OXPHOS) complexes (Fig 5). Complexes I (NADH dehydrogenase, NDUFB8), II (succinate dehydrogenase, SDHB), IV (cytochrome c oxidase, MTCO1), and V (ATP synthase, ATP5A) were increased by between 30 to 85% (p<0.05) when miR-194 was inhibited compared to control (Fig 5). Complex III (Q-cytochrome c oxidoreductase, QCRC2) was not different.

## Discussion

Growing evidence indicates an important role of miRNAs in metabolism and glucose homeostasis; thus miRNAs could be involved in the pathogenesis of type 2 diabetes. This study examined the role of microRNAs at different stages in the programming and progression of T2DM. The focus was on skeletal muscle which accounts for over 80% of glucose disposal in the body. The results of this study reveal an important role of miR-194 in influencing glucose metabolism in association with the progression from insulin resistance to type 2 diabetes. Indeed, we identified miR-194 as being down-regulated in skeletal muscle of insulin resistant rats from HF fed dams, high fat fed mice, and humans with pre-diabetes and T2DM. We investigated the functional role of miR-194 in skeletal muscle cells and showed that miR-194 down-regulation is associated with: 1) increased basal and insulin-stimulated glucose uptake, 2) increased glycolysis and incorporation into glycogen, 3) increased AKT and GSK3β phosphorylation in response to insulin stimulation, 4) increased glucose oxidation capacity, and 5) increased basal protein expression of mitochondrial oxidative phosphorylation complexes in skeletal muscle. Thus, miR-194 could be down-regulated in patients with early features of diabetes as an adaptive response to facilitate glucose metabolism in the face of insulin resistance. These findings provide mechanistic insight into the role of microRNA regulation of skeletal muscle glucose homeostasis.

### Insulin resistance alters the microRNA signature in skeletal muscle

The rapid increase in T2DM over the past decades has been caused by interactions between genetic susceptibility and environmental factors, such as unhealthy diet and sedentary lifestyle [21]. Epigenetics is now understood to be an important regulator during critical periods of development which can permanently alter phenotype. In particular, miRNAs have emerged as key regulators of gene expression [2]. They have also been implicated in the regulation of glucose and lipid metabolism and in diabetes [7, 8, 22–25]. To identify miRs involved in the susceptibility and progression to T2DM, we performed microRNA microarrays using a combined experimental and clinical approach involving: 1) a rat model of maternal high fat feeding during gestation and lactation that induces the development of insulin resistance in chow fed offspring in the absence of obesity, and 2) human skeletal muscle biopsies from patients across the spectrum from healthy to pre-diabetes and established type 2 diabetes.

Male offspring of HF dams demonstrated higher circulating insulin levels and, although their body weight was not different from controls, there was increased regional adiposity [15]. The development of insulin resistance is commonly seen prior to the development of T2DM [26], and has also been described in several models of maternal overnutrition [9]. The detrimental effects of overnutrition during fetal and early postnatal life (i.e. increased adiposity, hypertension, insulin resistance, and obesity have been supported by numerous studies in animal models [11, 12]. Patients with pre-diabetes or T2DM presented with abnormal fasting plasma glucose levels and/or abnormal glucose levels 2 hours after an oral glucose tolerance test (OGTT). Interestingly, in our human cohort, 4 out of 5 volunteers with pre-diabetes and 3 out of 6 volunteers with type 2 diabetes had a family history of T2DM.

Microarrays revealed an altered miR signature in the skeletal muscle of insulin resistant rats, patients with pre-diabetes and patients with T2DM. Among the miRs that were differentially expressed in the skeletal muscle of patients with PD/T2DM, and in the insulin resistant rat offspring compared to their controls, some have been previously described in the literature as linked to T2DM. For example, miR-206 and miR-29b are known to be significantly upregulated in the context of diabetes [27, 28] and such increase was confirmed in our human cohort.

Interestingly, a unique microRNA was regulated across both species: indeed, miR-194 expression was significantly reduced by 25 to 50% in both models. A negative correlation between miR-194 expression and the HOMA-IR also indicated an inverse association of this miR with insulin resistance. Moreover, we measured miR-194 expression in the skeletal muscle of high fat fed mice with established insulin resistance [18] and also showed a 37% decrease in the skeletal muscle of these animals. This experimental model has been found to induce whole-body insulin resistance which is initiated by impaired hepatic insulin action (after 1 week) and exacerbated by skeletal muscle insulin resistance (observed after 3 weeks) and is associated with the accumulation of specific bioactive lipid species [18]. A role for miR-194 in type 2 diabetes has not yet been described in the literature; however, Zhang J. *et al*. showed a decreased hepatic expression of miR-194 in the adult offspring of high fat fed dams [29]. Of note, we also found a decreased miR-194 expression in the adipose tissue from our human cohort (-21% in patients with pre-diabetes, -38% in patients with T2DM, data not shown).

*In silico* prediction of miRNA target genes is an important tool to determine the biological pathways regulated by miRNAs [30–32]. A computational prediction of miR-194 target genes has suggested several targets linked to T2DM signaling pathways (ATM, AKT2, KCNJ11, MAPK1, SOCS2), insulin signaling (AKT2, ATM, CRK, FOXO1, GRB10, INPP5K, MAPK1, PRKAR1A) and AMPK signaling (ADRAP1A, AKT2, ATM, CHRNAS, MAPK1, PPAT, PPP2R2C, PRKAR1A).

### miR-194 down-regulation promotes multiple aspects of glucose metabolism in skeletal muscle

When studying the effects of miR-194 inhibition in L6 myocytes, we found that down-regulation of miR-194 resulted in an increased glucose uptake into the muscle cells and incorporation into glycogen both basally and in response to insulin. The potentiation of glucose metabolism in response to insulin can be explained by upregulation of the insulin signaling pathway. Indeed, our study revealed augmented levels of insulin-stimulated phosphorylation of AKT and GSK3β by miR-194 inhibition. AKT is normally activated by insulin through phosphorylation of the Thr 308 and Ser 473 sites respectively [33]; then AS160 (AKT substrate of 160 kDa) is phosphorylated and inhibited. This induces the redistribution of GLUT-4 from intracellular vesicles to the plasma membrane, thus regulating glucose uptake [34]. AKT is also involved in the regulation of glycogen synthesis through the serine/threonine kinase glycogen synthase kinase 3 (GSK3). This enzyme consists of two isoforms, GSK3α and GSK3β, is inactivated through phosphorylation on stimulation and inactivates glycogen synthase (GS) by phosphorylation [35]. It has been shown that GSK3 is overexpressed and overactive in skeletal muscle in obesity and T2DM [36, 37] [38]. Moreover, exposure to maternal obesity during the periconceptional period results in the programming of changes to molecules downstream of AKT, GLUT-4 and GSK3 [39]. Thus, reduced miR-194 may elevate glucose uptake and its incorporation into glycogen by activating AKT and inactivating GSK3 through phosphorylation.

We have also shown an increased glycolysis in response to the down-regulation of miR-194, as indicated by an elevated lactate production in transfected cells. Interestingly, the absence of further increase of lactate production when transfected cells are treated with insulin may indicate that the glycolytic rate within miR-194 inhibited cells is already operating at maximal capacity.

Glucose oxidation rates were unaffected by miR-194 inhibition under basal conditions, but in the presence of the mitochondrial uncoupler FCCP, cells had an increased oxidative capacity. These data suggest a reduction in miR-194 may allow greater oxidation of substrate, specifically glucose, under periods of increased substrate supply or under metabolic stress where greater ATP turnover is required. Considering glucose oxidation was assayed under basal conditions, it might be interesting to determine whether miR-194 inhibition can increase glucose oxidation under simulated nutrient excess.

In line with our oxidative phenotype, L6 muscle cells transfected with a miR-194 inhibitor had higher protein expression of several complexes of the mitochondrial oxidative phosphorylation (OXPHOS) chain, a proxy measure for mitochondrial volume. A greater mitochondrial volume provides a likely explanation for the increase in oxidative capacity we observed in miR-194 silenced cells.

The tightly controlled process of mitochondrial OXPHOS, which involves 5 different protein complexes (Complex I-V), is responsible for the production of ATP. Deterioration of mitochondrial function is often present in metabolic diseases [40]; altered oxidative metabolism and mitochondrial structure, and impaired biogenesis have indeed been described in models of insulin resistance or T2DM [41, 42]. MiRs also play a role in the regulation of mitochondrial function, either under physiological or pathological conditions [43]. We have also shown that offspring of dams fed a high fat diet during pregnancy and lactation suffered from a decline in mitochondrial OXPHOS activity in adulthood [15]. Conversely, we observed that decreased expression of miR-194 for a short term (48 hours *in vitro*) is able to increase muscle mitochondrial oxidative capacity potential through increased mitochondrial OXPHOS. This may be an early compensatory mechanism to adjust to excess substrate supply and glycemia observed in metabolic disorders such as T2DM.

### Limitations

There is no gold standard for measuring miR expression; however, microarray and q PCR are the most recognized methods for evaluating miRs [44, 45]. Down-regulation of miR-194 identified by microarray was indeed validated by qPCR, showing good correlation between microarray and qPCR, and discarding the possibility of a false positive which can be high with microarray studies.

The microarray data from humans and rats provided a basis for the a priori hypothesis that miR-194 would be differentially expressed between the various models when assessed by qPCR.Though relatively small, the number of participants was nevertheless sufficient to detect significant and biologically relevant changes in muscle microRNA expression. Participants with either pre-diabetes or diabetes were obese (BMI≥30kg.m^-2^), while healthy participants had a BMI within the normal range. We cannot definitely determine whether the effects on skeletal muscle miR expression are driven by obesity or type 2 diabetes through cross-sectional comparisons of clinical data. However, through inclusion of an *in vivo* animal model where diabetes developed in the absence of obesity and also *in vitro* cell culture studies, we were able to further elucidate the relationship between miR expression and glucose metabolism. Our combined experimental and clinical approach allowed us to identify changes of miR-194 expression across multiple species and metabolic tissues (skeletal muscle, adipose tissue) at different stages of the disease progression suggesting that miR-194 may be involved in the initial cellular responses to hyperglycemia and be part of the early cellular events related to the pathogenesis of type 2 diabetes. Our functional experiments *in vitro* have provided strong evidence of the effects of miR-194 down-regulation on glucose metabolism; additional studies examining the effects of miR-194 inhibition *in vivo* are now warranted to confirm our data.

Likewise, functional studies should be carried on other microRNAs that showed significant changes in our human cohort or in our animal model. Evaluation of the roles of these miRNAs would provide a better understanding of their relative contribution to the pathogenesis of insulin resistance and diabetes.

### Translational potential

Epigenetics, in particular miRNAs, are considered important regulators of gene expression which can permanently alter phenotype. Though the understanding of the involvement of miRNAs in diabetes is in its infancy, advances in investigating the role of miRNAs in diabetes may potentially provide a powerful tool to predict, diagnose, treat, and prognose diabetes in the future [46].

Although we have not examined the mechanism by which miR-194 is down-regulated in skeletal muscle in the current study, it could involve selective packaging into exosomes. Measuring circulating miR-194 in the blood at different stages of the disease progression could inform on the potential of miR-194 as an early biomarker of diabetes, long before the onset of the disease. Indeed studies have demonstrated that miRNAs may act as potential biomarkers for diagnosis and prognosis of diabetes [47–51].

Changes in muscle metabolism associated with diabetes likely have multiple origins. While the aim of the current study was to identify miRs regulating glucose metabolism early in the history of metabolic disease, future studies which identify the complex interactions between miR expression and glucolipotoxic conditions in the context of metabolic disease are warranted. For instance, miR-194 expression was found increased in C2C12 cells treated with palmitate, and restored with oleate [52].

### Conclusions

In summary, our data indicate that miR-194 is down-regulated early in the development of insulin resistance and that this regulation is maintained through the progression towards type 2 diabetes. Furthermore, *in vitro* experiments showed miR-194 is involved in glucose uptake, glycolytic breakdown and its incorporation into glycogen, as well as mitochondrial oxidative capacity through mechanisms involving modulation of AKT, GSK3 and oxidative phosphorylation complexes. Taken together, our data suggest that the decrease in miR-194 expression observed in insulin resistant muscle is an adaptive response to facilitate tissue glucose uptake and metabolism in the face of insulin resistance. This aligns with the description of stress-responsive miRNAs that are regulated to maintain physiological processes [53, 54]. MiR-194 regulation thus appears to limit metabolic disturbances *in vitro*, yet its decrease *in vivo* is not sufficient to combat the progression to type 2 diabetes and the role of other key miRs warrants further investigation.
